# Comparative Study of Body Mass Index and Waist to Height Ratio of Korean Adults by Occupations

**Published:** 2017-10

**Authors:** Mi-Suk KIM, Tae-Kyum JUNG, Tae-Young KIM

**Affiliations:** 1. Dept. of Dance Arts, Convergence Culture and Arts, Sungshin Women’s University, Seoul, Korea; 2. Dept. of Taekwodo, College of Physical Education, Kyunghee University, Yongin, Korea; 3. College of Education, Hankuk University of Foreign Studies, Seoul, Korea

## Dear Editor-in-Chief

Traditionally, Body Mass Index (BMI) has been used for the studies of overweight and obesity. However, there is a limitation to apply it to all subjects; so waist to height ratio (WHtR) has begun to apply in central (abdominal) obesity studies. It is perceived as a much more accurate method not only for obesity but also for metabolic syndrome and chronic kidney disease patients (1); furthermore, WHtR is more efficient compared to BMI or waist circumference (WC) for heart disease patients (2). Because of measuring BMI and WHtR of 1010 (6^th^ Korean National Health and Nutrition Examination Survey, 2013–2014) adult males (3), there was a significant difference in body fat according to occupations (physical labor, mental labor) (4). The investigation results of the job strain and BMI of 52656 adult females showed that constant occupational stress appears to increase in proportion to the increase in BMI (5).

Health survey and medical checkup survey of The Sixth Korea National Health and Nutrition Examination Survey conducted the study. The total number of participants is 1770 (male: 1074, female: 696), and their occupations are classified as management position 391 (22.1%), professional occupation 290 (16.5%), office clerk 250 (14.2%), service 30 (1.4%), sales 184 (10.4%), agricultural and fisheries 82 (4.6%), and technicians 542 (30.8%); the investigated items were age, height, weight, BMI, waist circumference, WHtR of the subjects.

The collective grouping of obesity was taken place in accordance with the operational definition of the value of BMI and WHtR (6) matching status of BMI and WHtR and the frequency of occupation are shown in Table 1.

**Table 1:** Matching status of BMI and WHtR, and frequency of occupation

		***Matching Status of BMI and WHtR***					***Total***
		***a***	***b***	***c***	***d***	***e***	
Management Occupation group	Frequency (%)	2 (0.1)	48 (2.7)	30 (1.7)	31 (1.8)	280 (15.8)	391 (22.1)
Professional Occupation group	Frequency (%)	3 (0.2)	22 (1.2)	25 (1.4)	32 (1.8)	208 (11.8)	290 (16.4)
Office Clerk Group	Frequency (%)	3 (0.2)	18 (1.0)	21 (1.2)	18 (1.0)	190 (10.7)	250 (14.1)
Service Group	Frequency (%)	0 (0.0)	0 (0.0)	4 (0.2)	4 (0.2)	22 (1.2)	30 (1.7)
Sales Group	Frequency (%)	4 (0.2)	11 (0.6)	5 (0.3)	28 (1.6)	136 (7.7)	184 (10.4)
Agricultural and Fisheries Group	Frequency (%)	1 (0.1)	7 (0.4)	8 (0.5)	6 (0.3)	60 (3.4)	82 (4.6)
Technicians Group	Frequency (%)	11 (0.6)	50 (2.8)	74 (4.2)	25 (1.4)	383 (21.6)	543 (30.7)
Total	Frequency (%)	24 (1.4)	156 (8.8)	167 (9.4)	144 (8.1)	1279 (72.3)	1770 (100.0)

Pearson X^2^ = 62.451, df = 24 P=.000

low-BMI(1), normal-WHtR(2)=a, normal-BMI(2), low-WHtR(1)=b, normal-BMI(2), obesity-WHtR(3)=c, obesity-BMI(3), normal-WHtR(2)=d, BMI=WHtR=e

The value of all collected data was executed using SPSS version 18.0 (Chicago, IL, USA) to perform the mean and standard deviation, and a cross analysis (x^2^) was performed for the difference between the qualitative comparison group and the job.

In most of the occupations, matching groups (BMI=WHtR) were found to have a higher population, however, the mismatched groups (BMI≠WHtR) showed an even distribution across office clerical and service occupational groups. In addition, agricultural and fisheries groups showed an even distribution, and it appeared to be high in normal-BMI, obesity-WHtR group, which is group c (n=8, 0.5%). An even distribution of group a, b, c was shown in management group, yet, normal-BMI and low-WHtR, which are group b, was shown at highest level (n=48, 2.7%). Likewise, professional occupational group was shown the even distribution in group b and c, however, the obesity-BMI and normal-WHtR group, which is group d, were shown the highest level (n=32, 1.8%).

Sales group was found to be higher in obesity-BMI and normal-WHtR group, which is group d (n=28, 1.6%), while technical service group was found to be high in normal-BMI and obesity-WHtR group (n=74, 4.2%), however, in normal-BMI and low-WHtR group (n=50, 2.8%) which are group c and b, respectively.

Moreover, in service group that is the least number of subjects surveyed was shown high in normal-BMI and obesity-WHtR group which is group c, and also high in obesity-BMI and normal-WHtR group which is group d (n=4, 0.2%). BMI and WHtR method showed that 72.3% of the measurement was consistent in accordance with the jobs; however, 27.7% were inconsistent. Therefore, WHtR is more reliable than BMI for measuring body fat by occupation.
